# Correction: New cyclic glycolipids from *Silene succulenta* promote *in vitro* MCF-7 breast carcinoma cell apoptosis by cell cycle arrest and *in silico* mitotic Mps1/TTK inhibition

**DOI:** 10.1039/d3ra90085a

**Published:** 2023-09-06

**Authors:** Sarah A. Badawy, Ahmed R. Hassan, Rawah H. Elkousy, Salwa A. Abu El wafa, Abd El-salam I. Mohammad

**Affiliations:** a Medicinal and Aromatic Plants Department, Desert Research Center El-Matariya 11753 Cairo Egypt ahmedhassan@drc.gov.eg; b Department of Pharmacognosy, Faculty of Pharmacy (for Girls), Al-Azhar University Nasr City 11651 Cairo Egypt; c Department of Pharmacognosy, Faculty of Pharmacy (for Boys), Al-Azhar University Nasr City 13129 Cairo Egypt

## Abstract

Correction for ‘New cyclic glycolipids from *Silene succulenta* promote *in vitro* MCF-7 breast carcinoma cell apoptosis by cell cycle arrest and *in silico* mitotic Mps1/TTK inhibition’ by Sarah A. Badawy *et al.*, *RSC Adv.*, 2023, **13**, 18627–18638, https://doi.org/10.1039/D3RA01793A.

The authors regret that the name of one of the authors (Abd El-salam I. Mohammad) was shown incorrectly in the original article.

The Royal Society of Chemistry apologises for these errors and any consequent inconvenience to authors and readers.

## Supplementary Material

